# Effective unilateral/bilateral robot-assisted training for upper limb motor function rehabilitation: a cross-sectional study

**DOI:** 10.3389/fnhum.2025.1571624

**Published:** 2025-06-18

**Authors:** Guang Feng, Guohong Chai, Jiaji Zhang, Tao Song, Changcheng Shi, Jialin Xu, Guokun Zuo

**Affiliations:** ^1^Laboratory of Advanced Theranostic Materials and Technology, Ningbo Institute of Materials Technology and Engineering, Chinese Academy of Sciences, Ningbo, China; ^2^Key Laboratory of Mechanism Theory and Equipment Design of Ministry of Education, Tianjin University, Tianjin, China; ^3^Ningbo Cixi Institute of Biomedical Engineering, Ningbo, China

**Keywords:** rehabilitation robot, motor function recovery, training strategy, unilateral/bilateral training, multiple-modality feedback

## Abstract

**Objective:**

The therapeutic effect of robot-assisted training is still indecisive due to the lack of patient-tailored protocols and dose-matched training strategies when compared to traditional treatment. The objective of this study was to investigate the optimal robot-assisted training strategies for the upper limb functional recovery in hemiparetic stroke patients.

**Approach:**

A bilateral upper limb rehabilitation robot was employed to execute unilateral and bilateral training. Eighteen able-bodied subjects were recruited to test the effective of robot-assisted training strategies before transferring them to stroke patients. We compared unilateral passive training (UPT), bilateral passive training (BPT), and unilateral active training (UAT) with various feedback types (visual, force, and visual-force, none). These trainings were performed on three kinds of virtually-guided (straight-line, circular, S-shaped) tasks. Tracking error (TE), interactive force (IF) and target muscle activation level were quantified to characterize the motion capability and active participation of subjects.

**Main results:**

Results revealed that BPT-visual (0.63 ± 0.26) significantly increased muscle activation level when compared to those of BPT-none (0.45 ± 0.27) and UPT-visual (0.24 ± 0.05) (*p* < 0.01). UAT with single-modality feedback (visual/force) enabled higher TE (22.5 ± 3.40 mm) and active participation (0.78 ± 0.12) when compared with UAT with multi-modality (visual-force) feedback (TE: 6.6 ± 0.8 mm; activation level: 0.53 ± 0.13) (*p* < 0.01). The relatively complex circular and S-shaped tasks significantly enhanced the benefits of various training strategies.

**Significance:**

The current outcomes provide valuable guidelines for designing individualized robot-assisted training protocols, potentially promoting the clinical rehabilitation effect.

## 1 Introduction

Currently, about 80% of post-stroke patients suffer from upper limb motor impairment and neurological deficits, which have a serious impact on their quality of life (Rodgers et al., 2019). The theory of neuroplasticity holds that plenty of repetitive and task-oriented motor learning can promote the structural and functional restoration of the central nervous system, thereby recovering the motor dysfunction of limbs (Pekna et al., 2012; Kleim and Jones, 2008). However, traditional rehabilitation treatment cannot meet the requirements of the ever-increasing patients due to a severe shortage of physical therapists (Alrabghi et al., 2018). By contrast, robot-assisted rehabilitation has become a viable alternative due to its distinct advantages, such as, high-intensity repetitive training, personalized exercise programs, and objective assessment.

A series of end-traction (Miao et al., 2017; Lum et al., 2006; Hogan et al., 1992; Hesse et al., 2003) and exoskeletal (Nef et al., 2007; Pirondini et al., 2016; Zimmermann et al., 2023) upper limb rehabilitation robots have been developed to cater to different rehabilitation needs in different patients. Rehabilitation robots integrated with virtual reality (VR) can provide goal-oriented virtual task scenarios and assist the patients conduct a variety of functional rehabilitation exercises. MIT-MANUS that is regards as the first upper limb rehabilitation robot can assist or disturb the planar movements of an upper limb (Hogan et al., 1992). The Bi-Manu-Track system can conduct bilateral training on both upper limbs to reduce the patients’ spasticity and improve the motor control ability (Hesse et al., 2003). 7-DOF ARMin exoskeleton robot can provide shoulder-elbow-wrist cooperative training a unilateral upper limb (Nef et al., 2007). Multicenter randomized controlled trials have confirmed the efficacy of robot-assisted training in facilitating functional recovery for patients, but comparative trials have yet to demonstrate a statistically significant advantage of robot-assisted training over traditional rehabilitation treatment (Lo et al., 2010; Remy-Neris et al., 2021). Hence, it is essential to study how to improve the effectiveness of robot-assisted training, since it has become a cynosure in the realm of human-machine collaborative rehabilitation (Zanatta et al., 2022). The existing researches have confirmed that design of personalized training strategies through the combination of different training modes and tasks, development of assist-as-needed (AAN) control algorithm specially for patient individual characteristics and functional responses, and promotion of active participation from physical and psychological levels are the mission-critical tasks of patient-tailored robot-assisted rehabilitation (Brown and Xie, 2024).

Rehabilitation robots generally can provide multiple training modes, including passive training, active training, and resistance training. These modes are adopted for motor function rehabilitation of different degrees (severe or mild) of impaired limbs (Babaiasl et al., 2016). Robot-assisted passive training is usually designed based on the position control algorithm, but is hard to motivate the active participation of the patients. Therefore, kinds of AAN-based strategies are extensively proposed to enhance the effectiveness of the robot-assisted control and patients’ active participation (Li H. et al., 2022; Li M. et al., 2022; Sharifi et al., 2020; Luo et al., 2019; Pehlivan et al., 2015). The principle of AAN aims to mobilize the full potential of a patient’s active force by providing the minimal level (appropriate) of robot-assisted force. In addition, if the impaired limb is unable to perform/follow the pre-defined trajectories of a training task due to spasticity or severe motor dysfunction, a time-independent control method based on potential field constraints is proposed to constrain the patients’ training exercises within a certain error ranges relative to the target trajectories (Feng et al., 2022a; Najafi et al., 2020), therefore ensure the safety and reliability of rehabilitation training.

Multiple sensory feedback, including visual, auditory, and haptic sensations, can be used to attract a patient’s attention and encourage his/her active participation in the rehabilitation training (Daniel Ona et al., 2019). Visual feedback contains a wealth of detailed information about the shapes and movements of target training areas (Welch and Warren, 1980). Visually-guided feedback can effectively assist a patient to perform rehabilitation exercises following pre-defined target trajectories and simultaneously remind he/she to adjust the training status in time. Rhythmic auditory feedback usually provides an attentional cueing for repetitive training and keeps the limb movement in the same beats (Whitall et al., 2000). In addition, force feedback, which can enhance the sense of presence in VR (Minogue and Jones, 2006), is also adopted to help or disturb the execution of patients’ rehabilitation training. Moreover, some studies have demonstrated that multiple-modality feedback can improve the learning efficiency of training tasks when compared to single-modality feedback (Sigrist et al., 2015; Cuppone et al., 2018). Nevertheless, it might cause relatively higher mental burden on patients while complex multiple-modality feedback (e.g., hybrid visual-auditory-force feedback) is combined with different rehabilitation tasks, and then influence the effects of rehabilitation training. Therefore, in terms of robot-assisted upper limb rehabilitation, it is essential to investigate the interactive mechanism of the robot-assisted training modes, typical rehabilitation tasks and feedback types, since so far it has not been quantitatively evaluated on patients due to the lack of standardized protocol and optimal control groups (Ho et al., 2019; Nath et al., 2022). This is important and meaningful to improve the effect of robot-assisted training in practice.

In this study, we intended to verify that typical unilateral and bilateral training combined with optimal virtually-guided tasks and visual/force feedback can effectively improve the effects of the robot-assisted rehabilitation. To this aim, we conducted a quantitative evaluation of three common robot-assisted training modes unilateral passive training (UPT), bilateral passive training (BPT), and unilateral active training (UAT) using a bilateral upper limb rehabilitation robot. The study tested these modes across 18 able-bodied subjects, applying three virtually-guided tasks (straight-line, circular, S-shaped) and four feedback types (visual, force, visual-force, none). Only visual feedback is considered in the passive training. The TE, IF and target muscle activation of the subjects were used to comprehensively characterize the subjects’ manual control capability and quantify the interaction effects of the robot-assisted training mode, rehabilitation task and visual/force feedback. The effectiveness and utility of robot-assisted training were also surveyed through a questionnaire. The current findings provide insights into patient-tailored robot-assisted training and can be directly applied to clinical treatment of commercial rehabilitation robot.

## 2 Materials and methods

### 2.1 Rehabilitation training and evaluation system

**Figure 1:**
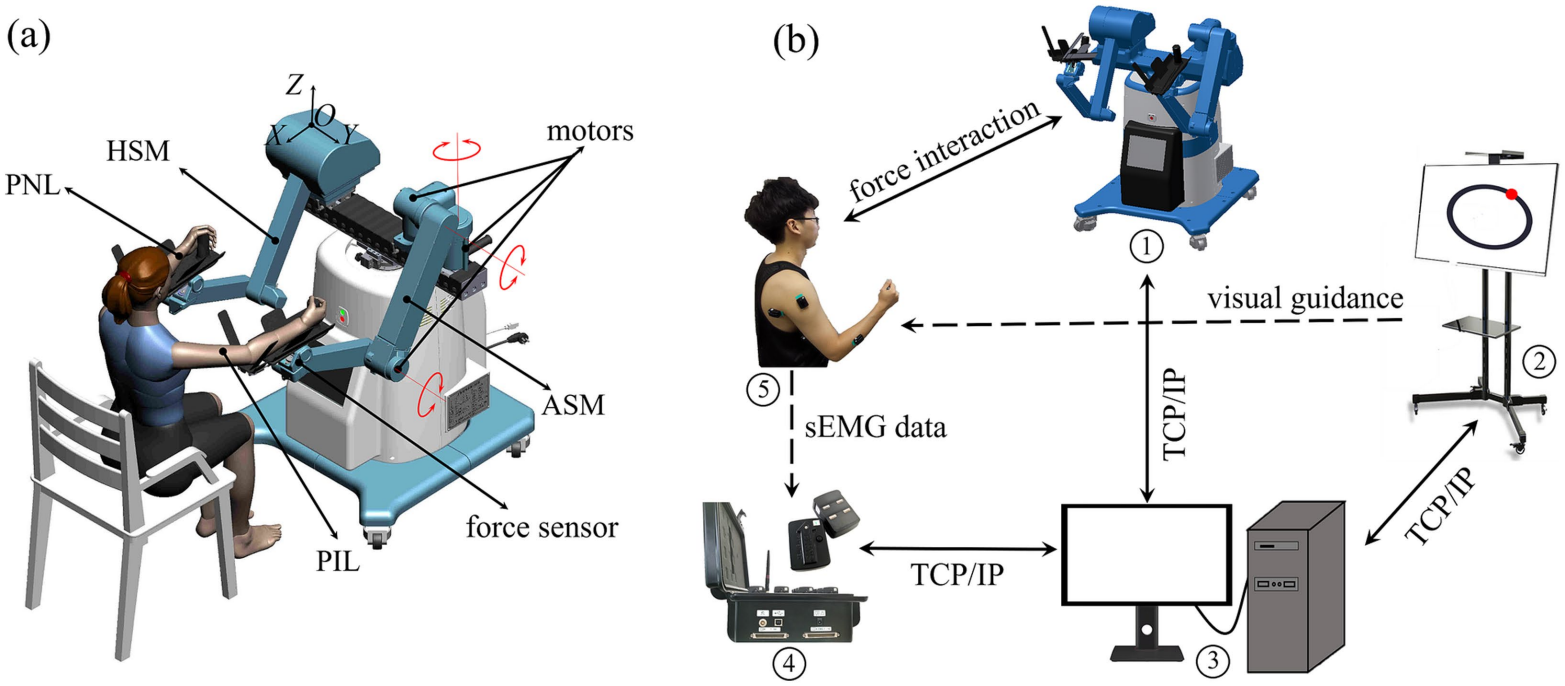
**(a)** The schematic diagram of bilateral upper limb rehabilitation robot (BULRR). **(b)** The experimental setup for robot-assisted rehabilitation training and evaluation.

A bilateral upper limb rehabilitation robot (BULRR) was developed and applied in this study (Feng et al., 2022b). The BULRR could perform unilateral/bilateral training as well as left and right limb coordination exercises. For post-stroke patients with unilateral limb dysfunction, the BULRR included an affected side manipulator (ASM) that interacted with a patient’s impaired limb (PIL), and a healthy side manipulator (HSM) that interacted with the patient’s normal limb (PNL). The ASM and HSM had identical mechanical structures, motors, and sensors. They could be driven by embedded motors or an external force, according to the requirements of robot-assisted rehabilitation tasks. The roles assignment of ASM and HSM depended upon the side of the PIL. In bilateral rehabilitation training, the ASM was passively driven by three motors, while the HSM was actively driven by the PNL. A schematic diagram of the BULRR is shown in Figure 1a. Figure 1b presents the integrated experimental setup for robot-assisted rehabilitation training and evaluation. It consisted of the following components: apart from the BULRR (①), a flat TV (②, Redmi X65T, Xiaomi Tech., China) was adopted to load and run the game development software (Unity3D 5.6.5., Unity Technologies, United States), and display the virtual rehabilitation task. To monitor a subject’s limb movement, the Delsys Trigno EMG System (④, Delsys. Inc., United States) was employed to acquire the sEMG signals of the target dominant muscles. A desktop (③, Dell, Intel Core i5-4590 3.3 GHz) was used to send control commands to the BULRR and the flat TV, and synchronously receive behavioral data from the BULRR and sEMG data from Delsys Trigno EMG system, respectively. The synchronous communication among the desktop, flat TV, and BULRR was implemented via TCP/IP protocol.

### 2.2 Virtual rehabilitation task and force feedback

**Figure 2:**
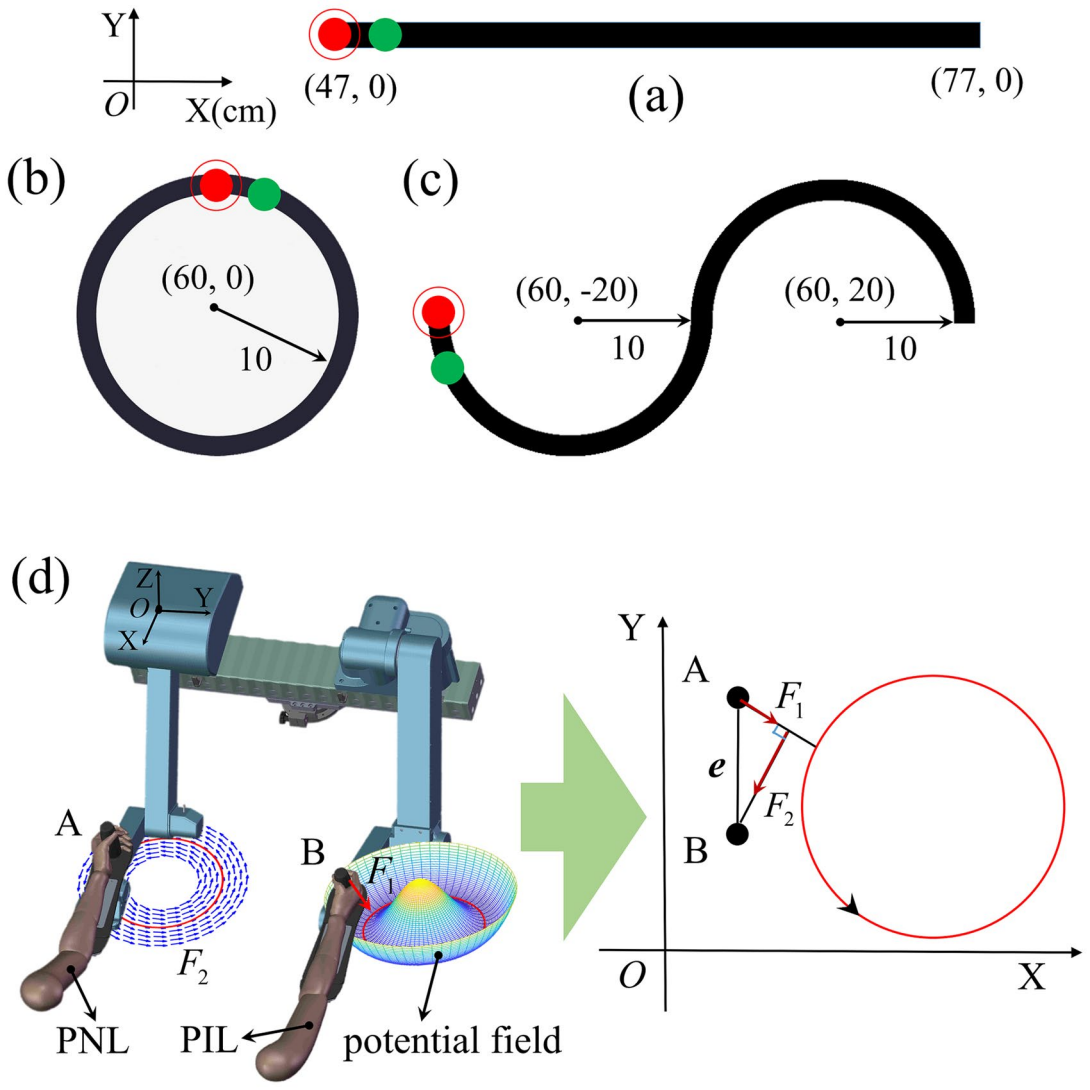
Visually-guided rehabilitation tasks used for guide the rehabilitation training trajectories, including **(a)** straight-line, **(b)** circular, and **(c)** S-shaped trajectory curves. The corresponding three target trajectories are located in the XOY plane (*Z* = 0) and their dimensions are labelled in the figure. The red and green balls are the agent points for endpoint B of PIL and endpoint A of PNL, respectively. The target trajectory is represented by black band. **(d)** Schematic diagram of bilateral passive training. Red circle signifies target trajectory. The normal force $F_{1}$ generated by the potential field is perpendicular to the target trajectory. Tangential force $F_{2}$ generated by position error ***e*** between A and B is parallel to the target trajectory. With the help of $F_{1}$ and $F_{2}$, the red ball tracks the green ball to complete the training task.

It has been proved that VR-based tasks can achieve immersive guidance and improve the performance of rehabilitation training (Calabrò et al., 2017; Howard, 2017). Hence, to enhance the subjects’ active engagement and the effects of interactive rehabilitation training, target-oriented virtual rehabilitation tasks were developed using Unity3D in this study. Corresponding to visually-guided functional requirements (Meng et al., 2023), we adopted three kinds of typical virtual rehabilitation tasks, i.e., straight-line, circular, and S-shaped visually-guided curves (target trajectories), to guide the subjects’ rehabilitation training, as depicted in Figures 2a–c. The red and green balls are the agent points characterize the real-time training trajectories of PIL and PNL, respectively. When the training starts, the red or green ball moves on the TV, and through visual feedback, the subject can see the position error between the actual trajectory and the target trajectory.

In addition, to keep safety and realize subject-specific rehabilitation training, task-oriented force feedback was integrated with the BULRR. The force feedback was applied through a potential field model (Feng et al., 2022b) according to the target training trajectory. As shown in Figure 2d, in the rehabilitation training, the normal force $F_{1}$ generated by the potential field prevented the endpoint of ASM deviating from the target trajectory in real time. The value of $F_{1}$ was positively correlated with the deviation distance from point A to the target trajectory. The position error ***e*** between points A and B was mapped to the force $F_{2}$, which drove the ASM parallel to the target trajectory. $F_{1}$ and $F_{2}$ worked together to assist a PIL to perform passive training. $F_{1}$ was used for UAT test, $F_{1}$ and $F_{2}$ were used for BPT test in this paper.

### 2.3 Rehabilitation training strategies

The BULRR could offer both unilateral and bilateral functional training in accordance with different upper limb rehabilitation demands of different patients. To investigate the effective rehabilitation strategies, three kinds of typical robot-assisted training modes were designed as follows. Note that PIL operates the ASM, and the PNL operates the HSM. In this paper, the subject’s right limb is defined as the PIL, and the left limb is defined as the PNL.

#### 2.3.1 Unilateral passive training

The PIL was fastened to the end of the ASM using straps, and the ASM could force the PIL to perform rehabilitation training, following pre-defined target trajectories. The patients do not need to apply any active effort during the unilateral passive training.

#### 2.3.2 Unilateral active training

The PIL was used to actively drag the ASM to conduct the preset rehabilitation training task. The ASM was already gravity and friction compensated. The used robot can set resistance levels to adjust UAT difficulty through a variable virtual damping *B* (*B* = 10 was adopted in the current study through a prior test).

#### 2.3.3 Bilateral passive training

This mode requires both PNL and PIL to be involved in the rehabilitation training. During the BPT, the patient was asked to use his/her PNL to actively manipulate the HSM to perform rehabilitation tasks, and the ASM was concurrently driven to drag the PIL to conduct identical passive rehabilitation training via synchronous/mirrored cooperative control of the BULRR, where the PIL is not actively exerting force.

### 2.4 Subjects

Eighteen able-bodied subjects (11 males and 7 females, mean age ± SD = 27 ± 7.6 years, mean height ± SD = 175 ± 7.4 cm, all right-handed) participated in the study. All experiments were conducted in accordance with the latest version of the Declaration of Helsinki and approved by the Ethics Committee of Human and Animal Experiments of Ningbo Institute of Materials Technology and Engineering, Chinese Academy of Sciences (Approval number: 2023C03160). All subjects were informed about the experimental procedure and signed the informed consent forms prior to participation.

### 2.5 Experimental protocol

#### 2.5.1 Experiment preparations

**Figure 3:**
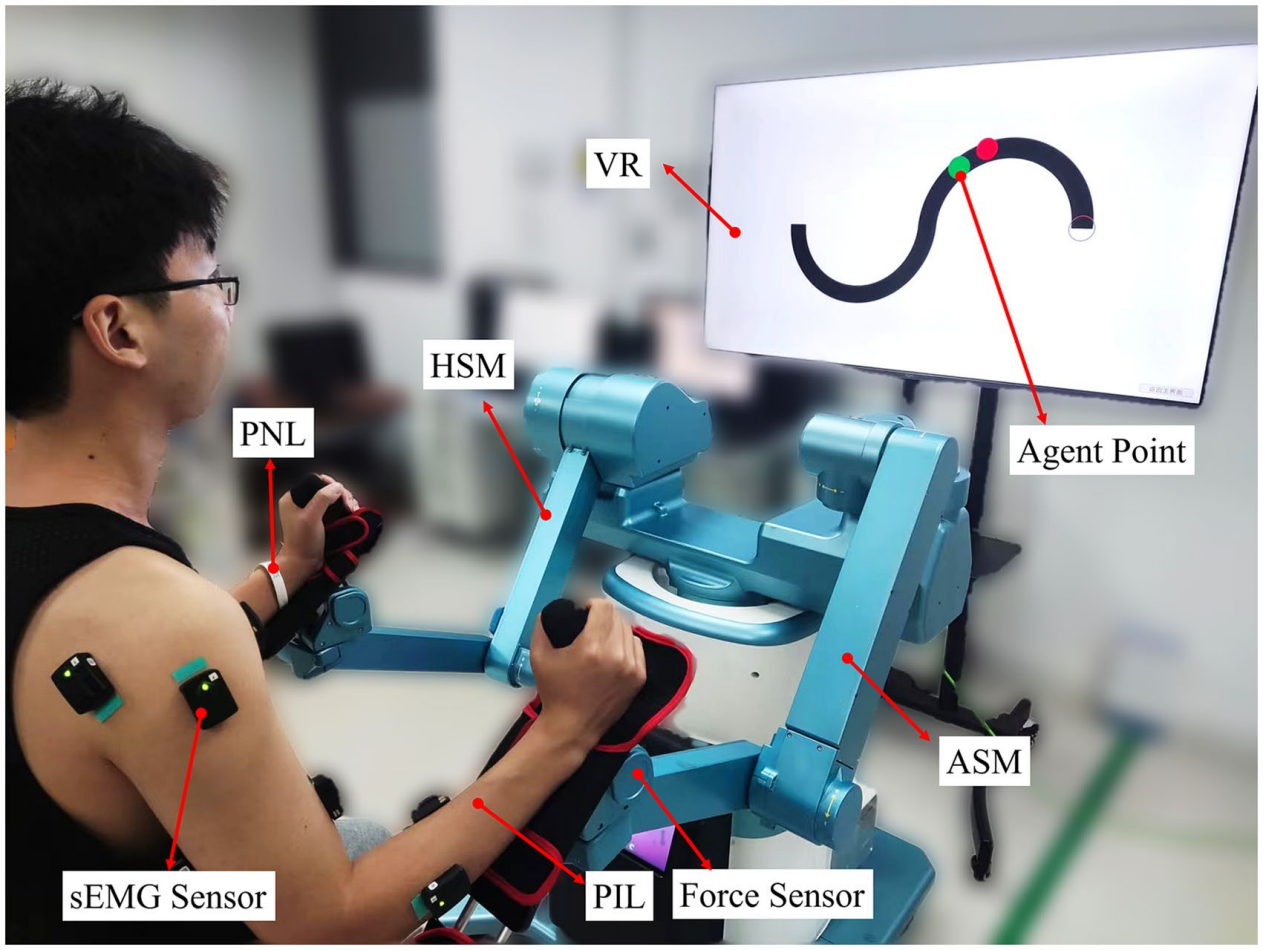
A subject was performing bilateral passive training on S-shaped trajectory with visual feedback.

The subject was seated in an upright position in a height-adjustable chair to test the rehabilitation effects of different robot-assisted training based on typical visually-guided tasks and multiple-modality feedback. According to the experimental requirements, the bilateral forearms of the subject were separately strapped onto the custom-made support brackets which were hinged with the ends of the ASM and AHM of BULRR. The subject could interact with the BULRR by operating two fixed handles of the support brackets in a comfortable position (see Figures 1a and 3). For all subjects, the right upper limb (right-handed) was assumed as the PIL, and the sEMG signals of key dominant muscles of PIL, including anterior deltoid, posterior deltoid, biceps, triceps, and radial wrist extensors were collected in the training task. Before mounting the sEMG sensors, all targeted skin areas were cleaned with alcohol pads to remove dirties and skin debris, to acquire high-quality sEMG data. Afterwards, three kinds of virtually-guided training tasks, the principle of operation and the experimental procedures were explained and the subject briefly practiced (3–5 min) manipulating BULRR, and started the formal experiment.

#### 2.5.2 Formal experiment

The formal experiment aims to test the rehabilitation effects of three kinds of training modes under three types of virtually-guided (straight-line, circular, S-shaped) tasks and four classes of feedback types (visual, force, visual-force, none). It included UPT, BPT, and UAT tests (see Table 1).

**Table 1:** Summary of robot-assisted training modes and virtually-guided tasks with multiple-modality feedback.

Rehabilitation training mode	Training task	Feedback type^a^	Number of repetitions
Unilateral passive training (UPT)	Straight-line	Visual	10
	Circular	Visual	10
	S-shaped	Visual	10
Bilateral passive training (BPT)	Straight-line	Visual/none	10
	Circular	Visual/none	10
	S-shaped	Visual/none	10
Unilateral active training (UAT)	Straight-line	Visual/force/visual-force/none	10
	Circular	Visual/force/visual-force/none	10
	S-shaped	Visual/force/visual-force/none	10

a Visual/force, visual-force and none are regarded as single-, combined-modality and no feedback, respectively.

##### 2.5.2.1 Unilateral passive training test

Subjects participated in the UPT with their PILs (right upper limbs). He/she was asked to perform UPT with three kinds of pseudo-random virtually-guided tasks (i.e., straight-line, circular, S-shaped) and visual feedback. A 5-min break was provided among different training tasks.

##### 2.5.2.2 Bilateral passive training test

Subjects used their PNLs to drive the PILs to complete the BPT. Each subject was required to perform three kinds of pseudo-random training tasks with or without visual feedback, successively. During the blind training tasks, the subject was blindfolded and his/her PNL was passively tracked target trajectories, followed by his/her PIL performing the training tasks under the guidance of the PNL.

##### 2.5.2.3 Unilateral active training test

The impacts of multiple-modality feedback on the UAT were investigated. Each subject was asked to complete three kinds of pseudo-random virtually-guided (i.e., circular, straight-line, S-shaped) tasks with visual, force, visual-force and none feedback, sequentially. The sequence of feedback conditions was intentionally structured to align with principles of motor learning and sensory integration. Similarly, in none feedback tasks, the subject was blindfolded and required to perform the training tasks by means of the pre-learning experiences from previous multiple-modality feedback tasks.

Each subject was asked to complete three kinds of training modes in sequence: UPT, BPT, and UAT. See Table 1 for the sequence of tasks. During the whole experiment, the breaks of 1–5 min were randomly given between different tests or different training tasks to allow the subjects to relax and physically recover. Every training task was repeated 10 times. The training periods of the three kinds of training tasks were set to 4.8 s for a straight-line task, 5 s for a circular task, and 10 s for an S-shaped task, depending on the lengths of respective target trajectories, respectively.

### 2.6 Data processing and evaluation metrics

#### 2.6.1 Interactive performance metrics

A force sensor (Figure 3) was used to measure the interaction force $F_{\text{inter}}$ between the PIL and the ASM in real time. The behavioral training data, including the interactive force $F_{\text{inter}}$, and the trajectories of training tasks were recorded and exported from BULRR. The terms of tracking error (TE) and interactive force (IF) were defined as evaluation metrics to assess the training effect. They were defined as


(1) $$\text{TE}=\frac{1}{N_{i}}\sum {\left\|T_{a}(i)-T_{t}(i)\right\|}_{2}$$

(2) $$\text{IF}=\frac{1}{N_{i}}\sum {\left\|F_{\text{inter}}(i)\right\|}_{2}$$

where $T_{a}(i)$ and $T_{t}(i)$ denoted the actual training trajectory and target trajectory of the *i*th (*i* = 1, 2…10) training trial, respectively. $N_{i}$ denoted the length of sampling points in each training trial. In the current study, with certain training modes and feedback types, the two kinds of metrics (TE and IF) of all trials in each training task of each subject were separately averaged as his/her TE and IF, and all subjects’ TE and IF were averaged as the mean TE and IF, respectively.

#### 2.6.2 Myoelectric activation index

The sEMG data were pre-processed as follows: the sEMG was removed mean, band-pass filtered with 5–500 Hz, then full-wave rectified, low pass filtered with a cutoff of 1.0 Hz. The processed data was used as eigenvalues of the sEMG (Potvin and Brown, 2004).

To further study users’ degree of active involvement, the root mean square (RMS) values of the five target dominant muscles were calculated to characterize the contribution ratio of muscle groups in the rehabilitation training (Jian et al., 2021).


(3) $$\text{RMS}=\sqrt{\frac{1}{n}\sum \text{sEMG}{\left(i,j\right)}^{2}}$$

where sEMG (*i*, *j*) was the preprocessed sEMG signals of the *j*th (*j* = 1, 2…5) muscle of the *i*th training trial, *n* represented the length of the sEMG signals in each training trial. RMS represented the activation level of the *i*th training trial in respective training tasks.

To quantify the activation levels of the selected five muscles across all subjects, all the RMS values of the respective five muscles in all training tasks of each subject were firstly normalized with min-max normalization, and then averaged across all subjects, according to respective training modes, tasks and feedback types (Table 1), respectively.

### 2.7 Psycho-physiological assessments

During the experiment, a psycho-physiological self-assessment was administered to every subject. Firstly, a NASA-TLX questionnaire (Hart and Staveland, 1988) on a scale from 0 (not at all) to 10 (completely) was conducted seven times to evaluate a subject’s mental burdens after each set of training (UPT/BPT/UAT) tests under feedback conditions, respectively. In addition, all subjects were asked to complete a brief evaluation questionnaire after the whole experiment. The questions, adapted from a previous study (Chai et al., 2017), probed the utility of unilateral/bilateral training, visually-guided task, and multiple-modality feedback, and whether it was feasible and applicable to subjects with different levels of upper limb dysfunction.

### 2.8 Statistical analysis

Statistical analysis was performed using IBM SPSS STATISTICS 22. Shapiro–Wilk test indicated that the data were not normally distributed. Thus, non-parametric tests (Kruskal–Wallis *H* test) were adopted to measure the significant influences of the passive training strategies (UPT-visual, BPT-visual, BPT-none), and training task (straight-line, circular and S-shaped) on the TE, IF and muscle activation levels, respectively. In the same way, Kruskal–Wallis *H* tests were adopted to measure the significant influences of the active training strategies (UPT), training task (straight-line, circular and S-shaped), and feedback type (visual/force /visual-force/none) on the TE, IF and muscle activation levels, respectively. Further pairwise comparisons were made using the Mann–Whitney test if necessary. A *p*-value <0.05 was considered statistically significant for all kinds of statistical analysis tests.

## 3 Results

Table 2 summarizes the mean TEs, IFs, activation level, and mean training time of three kinds of training tasks across all subjects in UPT, BPT, and UAT tests.

**Table 2:** Results of robot-assisted training modes and virtually-guided tasks with multiple-modality feedback.

Rehabilitation training conditions			TE (mm)	IF (N)	Activation level	Time (s)
Mode	Task	Feedback				
UPT	Straight-line	Visual	1.3 ± 0.0	5.6 ± 1.3	0.19 ± 0.13	4.8 ± 0.0
UPT	Circular	Visual	2.5 ± 0.3	5.8 ± 1.5	0.26 ± 0.14	5.0 ± 0.0
UPT	S-shaped	Visual	2.4 ± 0.3	6.2 ± 1.5	0.28 ± 0.13	10.0 ± 0.0
BPT	Straight-line	Visual	1.3 ± 0.0	5.4 ± 0.9	0.33 ± 0.10	5.0 ± 0.5
BPT	Straight-line	None	1.3 ± 0.0	6.8 ± 1.4	0.18 ± 0.11	4.4 ± 0.3
BPT	Circular	Visual	2.3 ± 0.2	6.1 ± 1.6	0.77 ± 0.13	5.2 ± 0.5
BPT	Circular	None	2.1 ± 0.3	5.3 ± 1.2	0.44 ± 0.12	4.6 ± 0.4
BPT	S-shaped	Visual	2.2 ± 0.1	6.0 ± 1.1	0.79 ± 0.11	11.3 ± 0.9
BPT	S-shaped	None	2.2 ± 0.1	6.2 ± 1.2	0.72 ± 0.11	9.5 ± 0.7
UAT	Straight-line	Visual	3.2 ± 0.3	7.4 ± 1.1	0.17 ± 0.08	3.7 ± 0.5
UAT	Straight-line	Force	4.9 ± 1.5	7.7 ± 1.1	0.13 ± 0.07	4.0 ± 0.5
UAT	Straight-line	Visual-force	3.2 ± 0.3	8.1 ± 1.3	0.10 ± 0.06	3.6 ± 0.4
UAT	Straight-line	None	5.2 ± 2.8	8.6 ± 1.3	0.12 ± 0.08	3.7 ± 0.4
UAT	Circular	Visual	11.8 ± 1.1	11.3 ± 1.0	0.78 ± 0.12	4.0 ± 0.3
UAT	Circular	Force	22.5 ± 3.4	14.5 ± 2.0	0.73 ± 0.10	4.0 ± 0.4
UAT	Circular	Visual-force	6.6 ± 0.8	8.8 ± 1.0	0.53 ± 0.13	3.5 ± 0.4
UAT	Circular	None	63.5 ± 10.0	11.9 ± 0.7	0.80 ± 0.08	3.7 ± 0.4
UAT	S-shaped	Visual	11.5 ± 0.9	9.5 ± 1.0	0.70 ± 0.14	8.1 ± 0.5
UAT	S-shaped	Force	18.5 ± 3.2	12.0 ± 1.1	0.69 ± 0.10	7.8 ± 0.6
UAT	S-shaped	Visual-force	6.0 ± 0.4	8.8 ± 0.7	0.49 ± 0.12	7.3 ± 0.6
UAT	S-shaped	None	50.9 ± 7.1	11.3 ± 1.2	0.68 ± 0.16	7.4 ± 0.6

### 3.1 UPT and BPT tests

**Figure 4:**
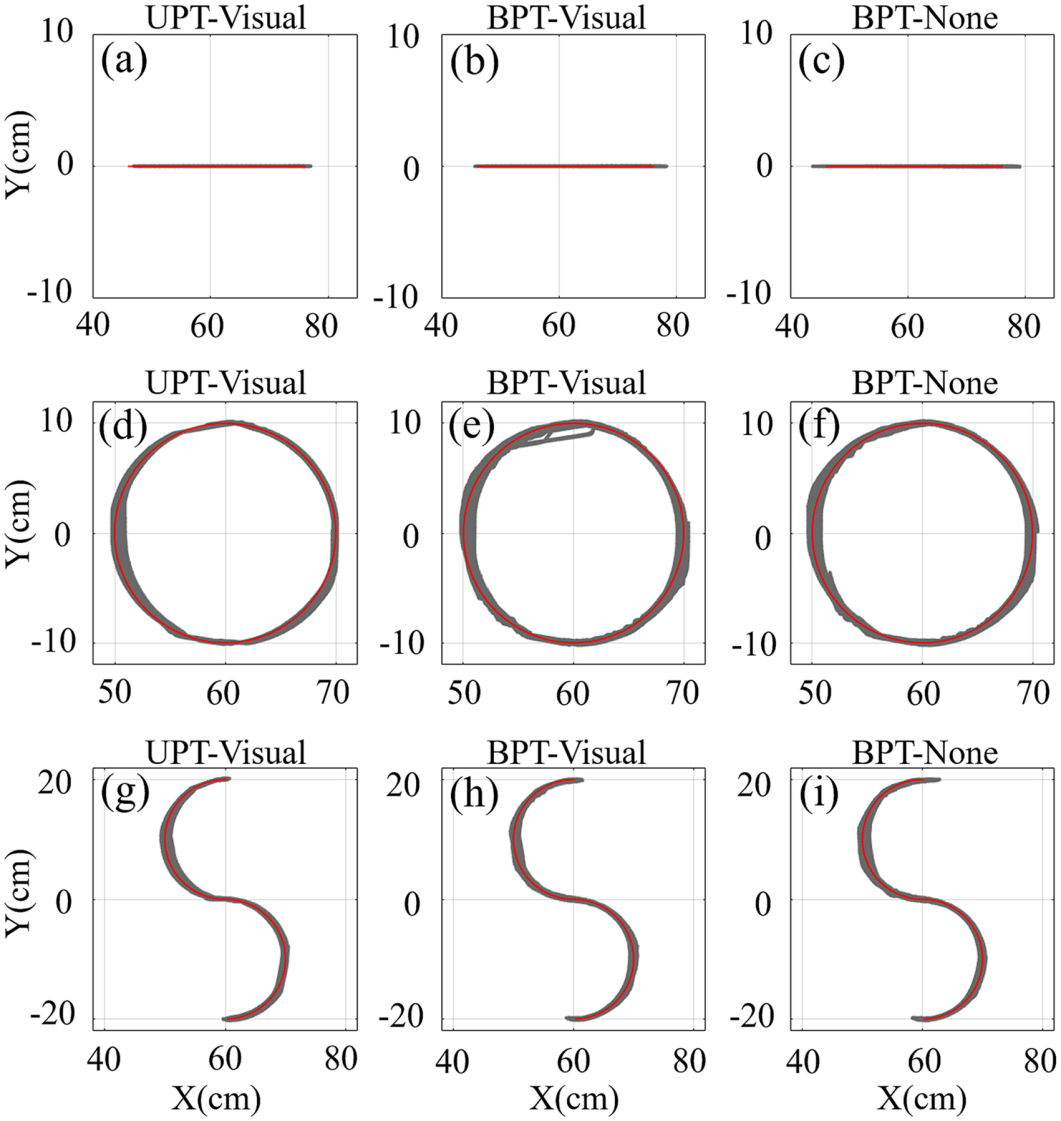
The trajectories of three kinds of training (straight-line, circular and S-shaped) tasks across all subjects (*N* = 18) in UPT and BPT tests with/without visual feedback. Red and gray lines/curves denote the target and training trajectories, separately.

The trajectories of three kinds of training (straight-line, circular and S-shaped) tasks across all subjects in UPT and BPT tests are shown in Figure 4. The results showed that both the unilateral and bilateral passive training modes led to similar performances in actual training trajectories. All subjects were able to complete three kinds of unilateral/bilateral passive training tasks well with or without visual feedback. No differences were observed among the UPT-visual, BPT-visual and BPT-none tasks, respectively.

**Figure 5:**
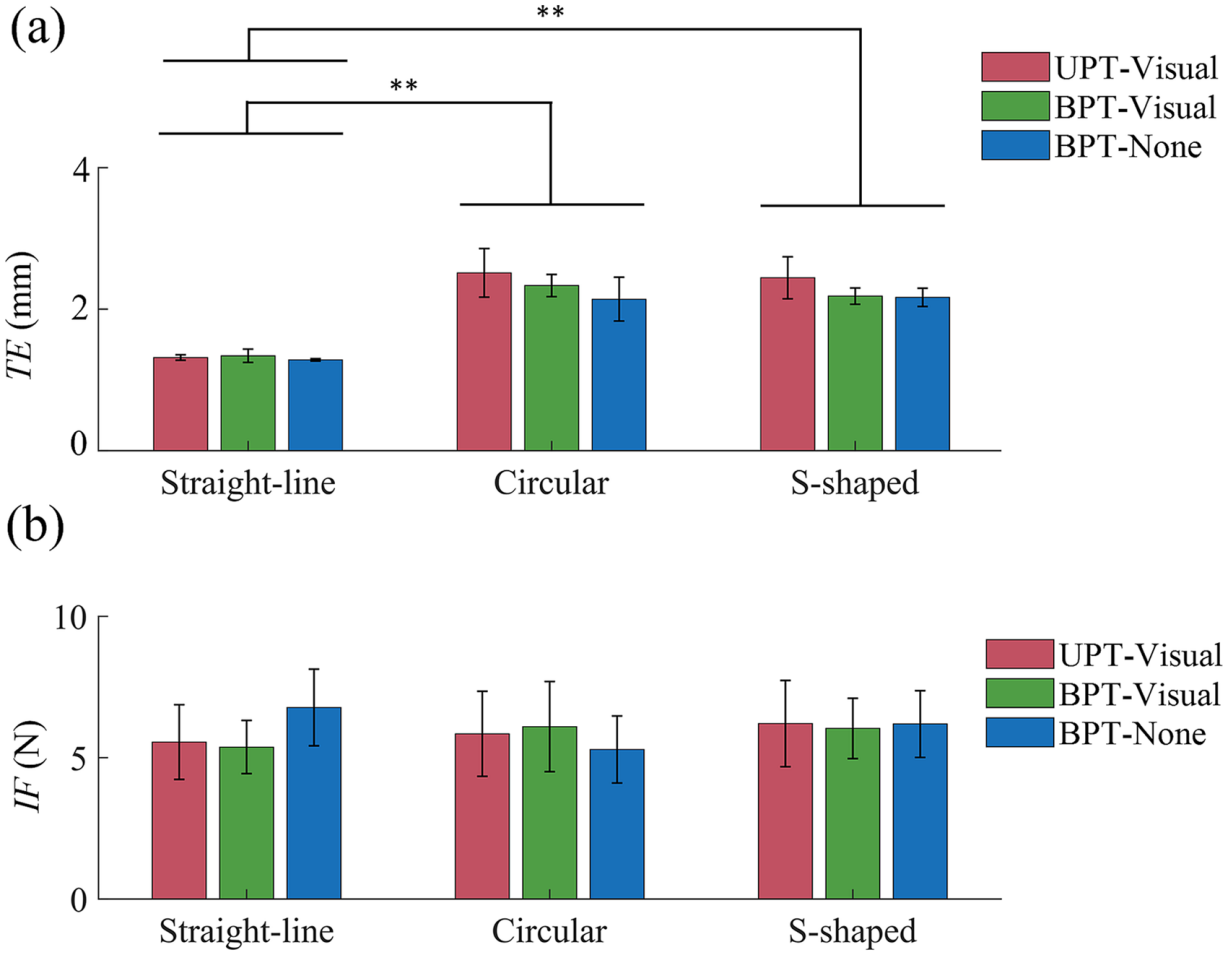
**(a)** Mean tracking errors (TEs) and **(b)** mean interactive force (IFs) of three kinds of training (straight-line, circular and S-shaped) tasks across all subjects in UPT and BPT tests. Symbols “*” and “**” indicate significant differences with a level of (*p* < 0.05) and (*p* < 0.01), respectively.

Figures 5a,b show the mean TEs (Equation 1) and IFs (Equation 2) of three kinds of training tasks across all subjects in UPT and BPT tests. Equation 3 is the definition that was used to quantify different muscle activation levels without adding citations. For conditions UPT-visual, BPT-visual and BPT-none, the mean TEs (1.3 mm ± 0.0 mm, 1.3 mm ± 0.0 mm, 1.3 mm ± 0.0 mm) with straight-line task were significantly less than those (2.5 mm ± 0.3 mm, 2.3 mm ± 0.2 cm, 2.1 mm ± 0.3 mm) with circular task (*p* < 0.01) and those (2.4 mm ± 0.3 mm, 2.2 mm ± 0.1 mm, 2.2 mm ± 0.1 mm) with S-shaped task (*p* < 0.01), respectively. Meanwhile, for conditions UPT-visual, BPT-visual and BPT-none, the Kruskal–Wallis *H* test indicated that no significant differences of the IFs were displayed among the straight-line (5.6 N ± 1.3 N, 5.4 N ± 0.9 N, 6.8 N ± 1.4 N), circular (5.8 N ± 1.5 N, 6.1 N ± 1.6 N, 5.3 N ± 1.2 N), and S-shaped (6.2 N ± 1.5 N, 6.0 N ± 1.1 N, 6.2 N ± 1.2 N) tasks, respectively.

**Figure 6:**
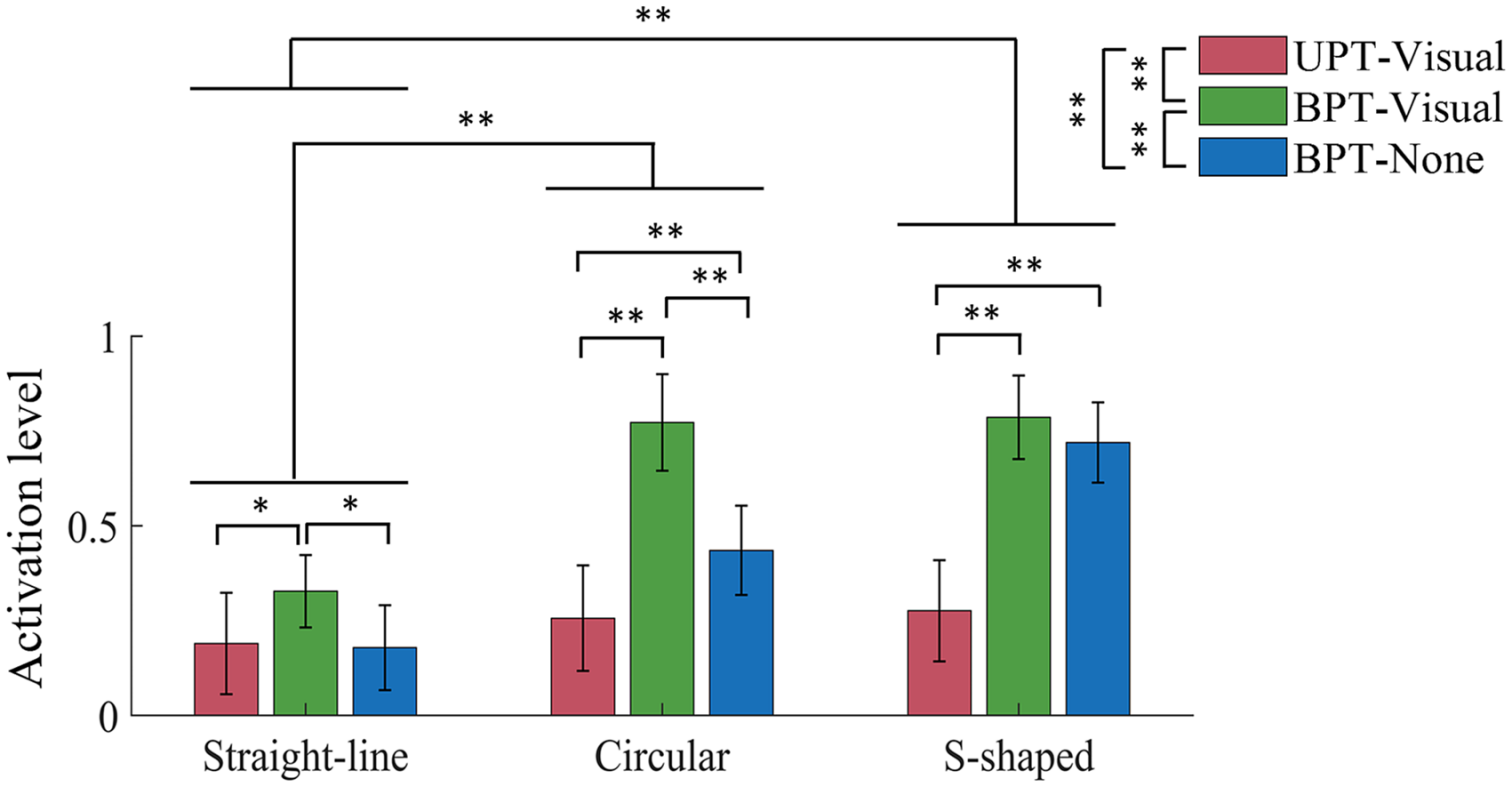
Mean normalization activation levels of five muscles across all subjects in UPT and BPT tests. All the acronyms and symbols are the same as those in Figure 5.

The normalized activation levels of the five target muscles across all subjects in UPT and BPT tests are shown in Figure 6. For training conditions UAT-visual, BPT-visual and BPT-none, the results showed that the overall muscle activation levels (0.19 ± 0.13, 0.33 ± 0.10, 0.18 ± 0.11) in straight-line task were significantly lower than those (0.26 ± 0.14, 0.77 ± 0.13, 0.44 ± 0.12) in circular task (*p* < 0.01) and those (0.28 ± 0.13, 0.79 ± 0.11, 0.72 ± 0.11) of S-shaped task (*p* < 0.01), but no significant difference displayed between the circular and S-shaped tasks, respectively. The Kruskal–Wallis *H* test indicated that there were significant differences among the three kinds of training conditions (UPT-visual, BPT-visual and BPT-none) (*p* < 0.01). Mann–Whitney *U* tests showed that there were significant differences of the activation levels within the respective training tasks with the exceptions of two pairwise comparisons in straight-line (UPT-visual versus BPT-none) and S-shaped (BPT-visual versus BPT-none) tasks, separately.

### 3.2 UAT test with multiple-modality feedback

**Figure 7:**
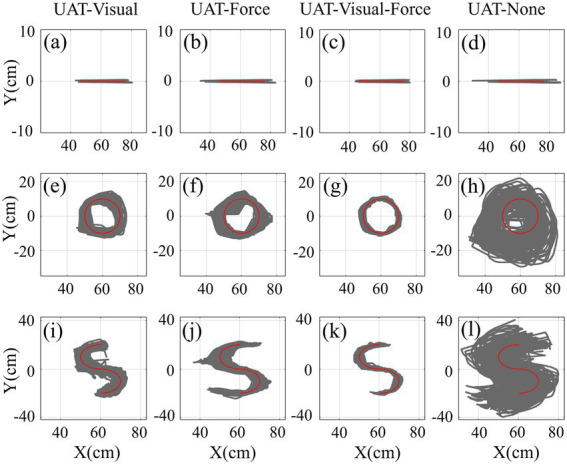
The trajectories of three kinds of training (straight-line, circular and S-shaped) tasks with four types of training conditions (UAT-visual, UAT-force, UAT-visual-force, UAT-none) across all subjects in UAT test. Representations of the lines/curves are the same as those in Figure 4.

The training trajectories across all subjects in UAT test are shown in Figure 7. The results displayed that the active training trajectories were obviously more scattered than the passive training trajectories (Figures 4, 7). For three kinds of training tasks, visual and force feedback could effectively help the subjects to track the target trajectories when compared to none feedback. Furthermore, the scatters of training trajectories with multiple-modality (visual-force) feedback were relatively greater than those with single-modality (visual or force) feedback. In particular, the subjects had difficulty performing the relatively complex (i.e., circular and S-shaped) rehabilitation tasks when sensory feedback was unavailable.

**Figure 8:**
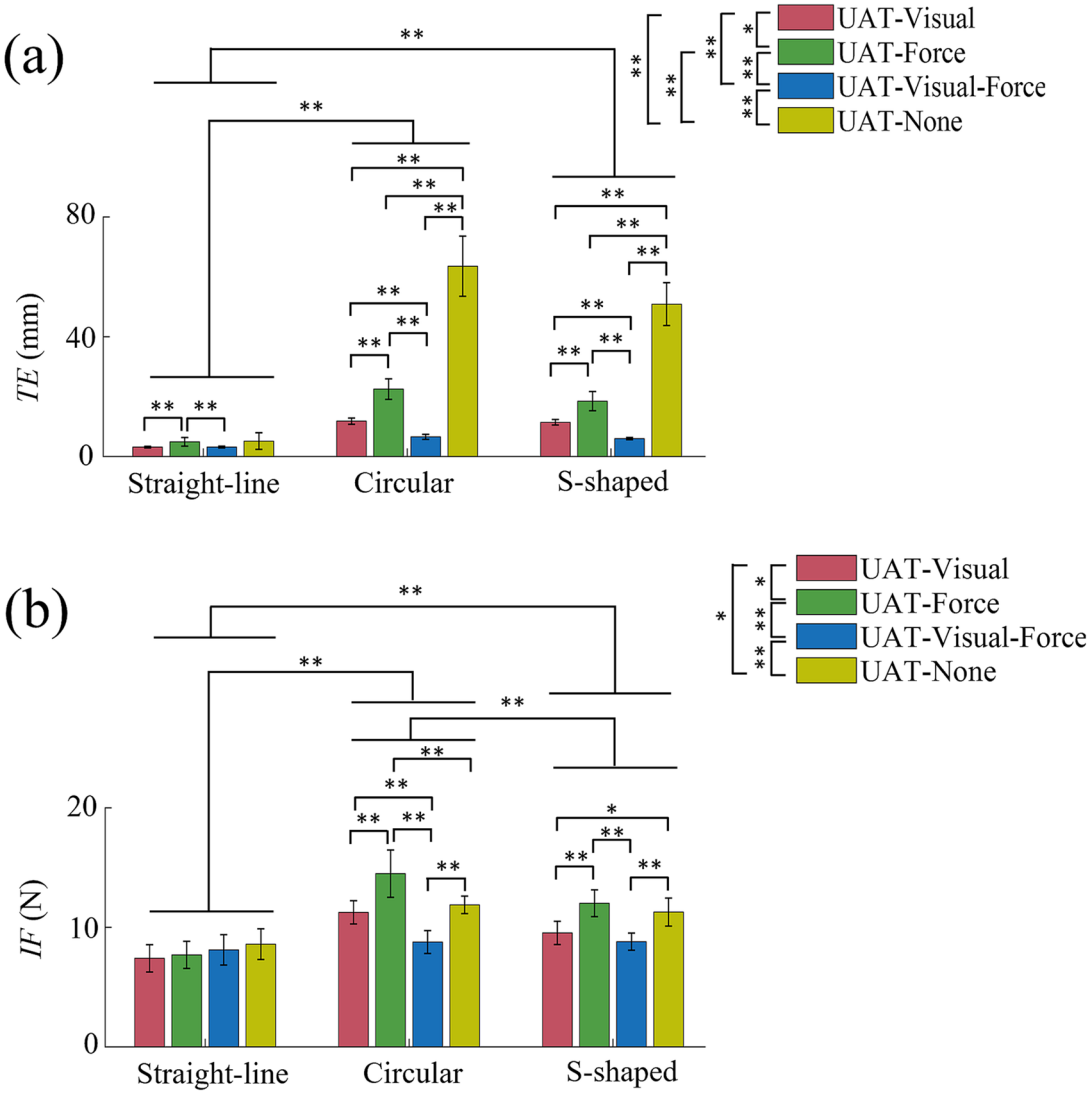
**(a)** Mean tracking errors (TEs) and **(b)** Mean interaction forces (IFs) of three kinds of training (straight-line, circular and S-shaped) tasks across all subjects in UAT test. All the acronyms and symbols are the same as those in Figures 5, 6.

Figure 8a shows the mean TEs of three kinds of training tasks across all subjects in UAT test, respectively. For training conditions UAT-visual, UAT-force, UAT-visual-force and UAT-none, the results revealed that the overall TEs (3.2 mm ± 0.3 mm, 4.9 mm ± 1.5 mm, 3.2 mm ± 0.3 mm, 5.2 mm ± 2.8 mm) in straight-line task were significantly smaller than those (11.8 mm ± 1.1 mm, 22.5 mm ± 3.4 mm, 6.6 mm ± 0.8 mm, 63.5 mm ± 10.0 mm) of circular (*p* < 0.01) and those (11.5 mm ± 0.9 mm, 18.5 mm ± 3.2 mm, 6.0 mm ± 0.4 mm, 50.9 mm ± 7.1 mm) of S-shaped (*p* < 0.01) tasks, but no significant difference of the TEs displayed between the two kinds of training tasks (circular versus S-shaped). The Kruskal–Wallis *H* test manifested that there were significant differences (*p* < 0.01) among the four types of training conditions, respectively, with the exceptions of one pairwise comparison (UAT-visual versus UAT-force, *p* < 0.05). Mann–Whitney *U* tests indicated that there were significant differences of TEs among the four types of training conditions within circular (*p* < 0.01) and S-sharped (*p* < 0.01) tasks, while only two pairwise comparisons (UAT-visual versus UAT-force, UAT-force versus UAT-visual-force) appeared differences (*p* < 0.01) in straight-line task.

Figure 8b shows the mean IFs of three kinds of training tasks across all subjects in UAT test, respectively. For training conditions UAT-visual, UAT-force, UAT-visual-force and UAT-none, the results displayed that the overall IFs (7.4 N ± 1.1 N, 7.7 N ± 1.1 N, 8.1 N ± 1.3 N, 8.6 N ± 1.3 N) in straight-line task were significantly smaller than those (11.3 N ± 1.0 N, 14.5 N ± 2.0 N, 8.8 N ± 1.0 N, 11.9 N ± 0.7 N) of circular (*p* < 0.01) and those (9.5 N ± 1.0 N, 12.0 N ± 1.1 N, 8.8 N ± 0.7 N, 11.3 N ± 1.2 N) of S-shaped (*p* < 0.01) tasks, and significant difference of the IFs also displayed between the circular and S-shaped tasks (*p* < 0.01). The Kruskal–Wallis *H* test manifested that there were significant differences among the four types of training conditions (UAT-visual versus UAT-force, *p* < 0.05; UAT-force versus UAT-visual-force, *p* < 0.01; UAT-visual-force versus UAT-none, *p* < 0.01; UAT-visual versus UAT-none, *p* < 0.05). Mann–Whitney *U* tests indicated that there were no significant differences of IFs among the four types of training conditions within straight-line task, while only a pairwise comparison (UAT-visual versus UAT-none) in circular task and two pairwise comparisons (UAT-visual versus UAT-visual-force, UAT-force versus UAT-none) in S-shaped task did not exhibit significant differences.

**Figure 9:**
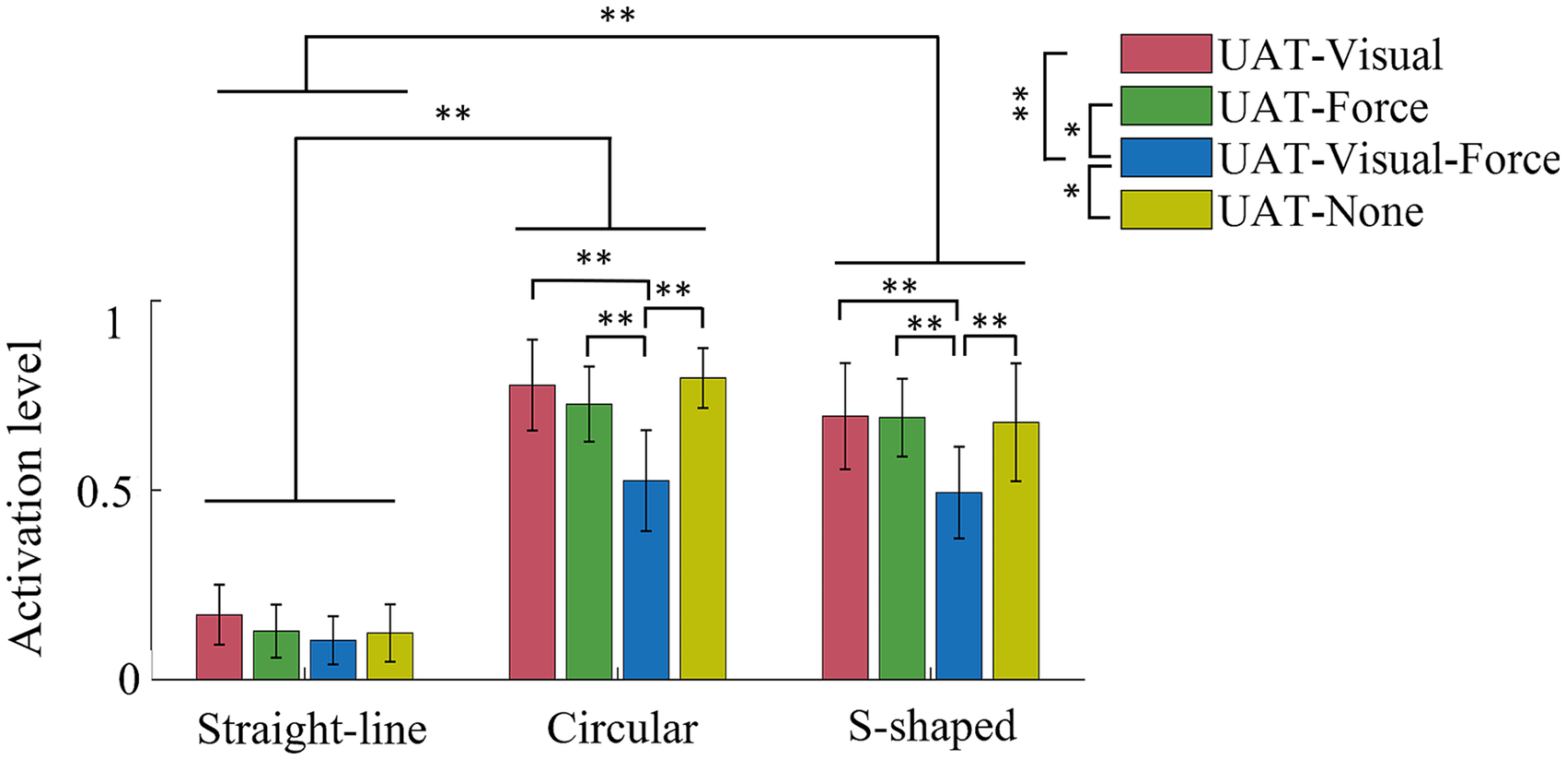
Mean normalization activation levels of five muscles across all subjects in UAT test. All acronyms and symbols are the same as those in Figures 5, 6, 8.

The normalized activation levels of the five muscles in UAT test are shown in Figure 9. For training conditions UAT-visual, UAT-force, UAT-visual-force and UAT-none, the results manifested that the overall activation levels of the five muscles (0.17 ± 0.08, 0.13 ± 0.07, 0.10 ± 0.06, 0.12 ± 0.08) in straight-line task were significantly lower than those (0.78 ± 0.12, 0.73 ± 0.10, 0.53 ± 0.13, 0.80 ± 0.08) of circular (*p* < 0.01) and those (0.70 ± 0.14, 0.69 ± 0.10, 0.49 ± 0.12, 0.68 ± 0.16) of S-shaped (*p* < 0.01) tasks, but no significant difference showed between the circular and S-shaped tasks. The Kruskal–Wallis *H* test showed that significant differences displayed among three pairwise training conditions UAT-visual versus UAT-visual-force (*p* < 0.01), UAT-force versus UAT-visual-force (*p* < 0.05), UAT-visual-force versus UAT-none (*p* < 0.05). Mann–Whitney *U* tests showed that there were no significant differences of the activation levels among the four types of training conditions within straight-line task, while three pairwise comparisons (UAT-visual versus UAT-visual-force, UAT-force versus UAT-visual-force, UAT-visual-force versus UAT-none) in circular task and three pairwise comparisons (UAT-visual versus UAT-visual-force, UAT-force versus UAT-visual-force, UAT-visual-force versus UAT-none) in S-shaped task displayed significant differences (*p* < 0.01), respectively.

### 3.3 Evaluation of questionnaires

**Figure 10:**
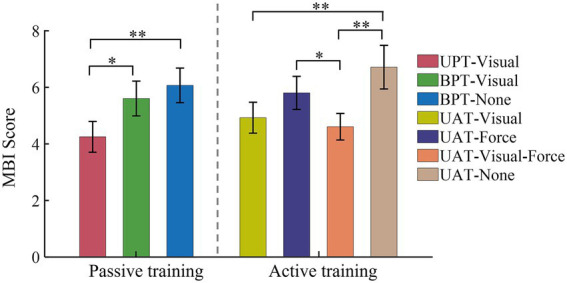
MBI scores of seven kinds of training conditions based on three kinds of typical training tasks. All acronyms and symbols are the same as those in Figures 5, 6, 8, 9.

The mental burden index (MBI) scores across all subjects in all rehabilitation training tests are shown in Figure 10. The MBI scores were averaged based on three kinds of typical training tasks with seven kinds of training conditions. In passive training tests, the MBI score (4.3 ± 1.1) on UPT-visual was significantly lower than that (5.6 ± 1.2) of BPT-visual (*p* < 0.05) and that on (6.1 ± 1.2) in BPT-none (*p* < 0.01), separately. In active training tests, based on training conditions UAT-visual, UAT-force, UAT-visual-force, and UAT-none, the MBI scores were 4.9 ± 1.1, 5.8 ± 1.2, 4.6 ± 0.9, 6.7 ± 1.5, respectively. The Mann–Whitney test showed that significant differences were displayed between the UAT-visual and UAT-none (*p* < 0.01), UAT-force and UAT-visual-force (*p* < 0.05), UAT-visual-force and UAT-none (*p* < 0.01), respectively.

Furthermore, all subjects reported that the current training strategies (seven types of training conditions) with typical (straight-line, circular and S-shaped) training tasks could be applied to the patients with different levels of upper limb dysfunction. All subjects expressed that UAT-visual-force could decrease the execution difficulty of training tasks. Four subjects (4/18) stated that BPT aroused an acceptable execution difficulty, and they (18/18) agreed that the current training strategies and training tasks were essential to robot-assisted upper limb functional rehabilitation.

## 4 Discussion

This study sought to demonstrate that commonly-used robot-assisted training with optimal virtually-guided tasks and visual/force feedback could be applicable to individual upper limb motor function rehabilitation. The results of training tests revealed that unilateral and bilateral training strategies with single- or combined-modality feedback enabled the subjects to perform typical rehabilitation tasks at relatively low TEs and high levels of active participation. These outcomes indicate the effectiveness of robot-assisted training being used for functional recovery in patients with upper limb dysfunction.

### 4.1 Comparisons of unilateral/bilateral training

Unilateral and bilateral training modes are abundantly employed in rehabilitation robots to recover the damaged upper limb and nerve functions of post-stroke patients via extensive repetition exercises (Renner et al., 2020; Xu et al., 2022). The functional impairment could be improved by activating the damaged hemisphere through coordinated movement between the most impaired arm and the less impaired arm (Cauraugh and Summers, 2005; Barber et al., 2008). However, numerous related literatures indicated that it was difficult to compare the effectiveness of the bilateral training and unilateral training due to no sufficient clinical results so far (Renner et al., 2020; Sheng et al., 2016; Chen et al., 2022). Both kinds of training modes had different functional roles according to different stroke phases of patients, training tasks and protocols (Chen et al., 2022). In the current study, we investigated the differences of three kinds of frequently-used training modes (UPT/BPT/UAT) based on consistent training tasks and visual/force feedback (Table 1), in combination with training trajectories, interactive forces and target muscle activation levels. The quantified results showed that the mean TEs in UAT (Figures 7, 8a) were obviously greater than those of two kinds of passive training modes (UPT and BPT) (Figures 4, 5a), but no significant differences displayed between UPT and BPT (Table 2). It was probably because the subjects had to perform the training tasks relying on their own upper limb motor functions under active training mode, while just needing to passively follow the predefined training trajectories both in unilateral/bilateral passive training modes.

The results of IF showed that the mean IFs in UAT test (Figure 8b) were obviously greater than those of two kinds of passive training modes (UPT and BPT) (Figure 5b). It indicated that the subjects put more efforts under active training mode when compared to unilateral/bilateral passive training modes. Due to the subjects’ PILs did not actively interact with the robot, there was no significant difference of the mean IFs between UPT and BPT (Table 2). However, the overall levels of muscle activation in the BPT test were significantly higher than that of the UPT (Figure 6), which was in accordance with that the both hemispheres of the brain were activated when subjects performed BPT (Renner et al., 2020). Furthermore, the muscle activation level on BPT-visual was significantly higher than that of the BPT-none (Figure 6), indicating integration of visual feedback could effectively promote the limb function rehabilitation. BPT with visual feedback might be more useful than unilateral training under the same conditions (Whitall et al., 2000). Therefore, it suggests that despite UPT and BPT modes can regulate goal-oriented robot-assisted training with slight TEs that are indispensable for patients with severe motor dysfunctions, the active training mode is crucial to the efficiency and effects of rehabilitation training (Luo et al., 2019). Nevertheless, the difference between the UPT and BPT modes is worth in-depth study by considering mirror-symmetrical bilateral training (Sheng et al., 2016).

### 4.2 Influences of multiple-modality feedback

Multiple-modality (e.g., visual/force or visual-force) feedback is crucial to robot-assisted rehabilitation training. It not only can provide necessary cues for patients to regulate the rehabilitation exercises, but also can potentially increase the effectiveness of rehabilitation training due to multi-sensory brain functional activation (Yuan et al., 2021). How to combine task-directed visual feedback and force feedback to improve the effects of rehabilitation training is an ongoing concern in the field of robot-assisted rehabilitation (Zheng et al., 2023). Our comparative results indicated that the subjects visual could hardly complete the relatively complex (circular and S-shaped) UAT tasks without sensory feedback (Figures 7h,l). Either kind of feedback was able to assist the subjects to regulate the training trajectories, and the combined-modality (Figures 7g,k) feedback could effectively decrease the TEs when compared to single-modality feedbacks (Figures 7e,f,i,j).

The Figures 8, 9 can further interpret the influences of the two kinds of feedbacks on the rehabilitation training. Based on circular and S-shaped tasks, the TEs on condition UAT-visual were significantly smaller than those of the UAT-force (Figure 8a). It indicates that target-oriented visual feedback enables the subjects to adjust training trajectories more easily than force feedback. This might be due to visual information could immediately response to the discrepancies of a training task, which helped them adjust their training trajectories in advance (Welch and Warren, 1980). Whereas, the condition UAT-force exhibited higher IFs than those of UAT-visual (Figure 8b). This was consistent with the training mode of force feedback, where real-time feedback force was positively associated with the magnitudes of TEs, preventing the subjects from deviating the target trajectories (Minogue and Jones, 2006; Feng et al., 2022b). Appropriate force feedback could effectively assist the subjects to perform the rehabilitation tasks (Zuo et al., 2024), especially for patients with visual impairment.

In contrast to single-modality feedback, combining visual and force feedback enabled the subjects to complete the training tasks with the minimal TEs and IFs (Figures 8a,b, circular, S-shaped tasks), as well as the minimal muscle activation levels (Figure 9). It can be interpreted that the multiple-modality feedback allowed the subjects to accurately regulate the training trajectories with less efforts (Sigrist et al., 2015). The relatively redundant feedback information can reduce the execution difficulties of training tasks and therefore decrease the activation levels of the target muscles. However, in terms of a post-stroke patient’s rehabilitation, the physical condition, degree of limb dysfunction and the task difficulties should be comprehensively considered in the selection of single-or combined-modality feedback.

### 4.3 Differences of virtually-guided tasks and questionnaires

The VR-based rehabilitation has shown its clinical potential for improving upper limb function and independent activities of daily living (ADL) in post-stroke patients when compared to intensity-matched traditional rehabilitation (Ahn and Hwang, 2019; Nath et al., 2022). Nevertheless, the optimal therapeutic effect of VR is still indecisive, owing to the lack of patient-tailored VR-based rehabilitation tasks, standardized protocol, optimal control group or quantifiable metrics to evaluate its effectiveness (Ho et al., 2019; Nath et al., 2022). Therefore, three kinds of visually-guided training tasks were compared based on consistent training modes and feedback conditions. The experimental results displayed that compared to straight-line task, the circular and S-shaped tasks led to more obvious TEs and target muscle activation in passive training mode (Figures 5a, 6), and TEs, IFs and muscle activation in active training mode (Figures 8, 9), respectively. While no significant differences appeared between the circular and S-shaped tasks (except for IFs in active mode). It suggests that the relatively complex visually-guided tasks can enhance the benefits of training rehabilitation. However, in terms of patients with limb dysfunction, design of visually-guided training tasks should not only consider its guiding functions, but also a patient’s actual condition (physical and neurological impairments) and his/her motor control ability. Moreover, hand-elbow-shoulder should be regarded as a motion whole to reflect the actual motor rehabilitation state of patients (Jiang et al., 2023). The patient-specific serious game-based rehabilitation tasks are further required to enhance the actual rehabilitation effect.

The differences of robot-assisted training strategies also can be manifested by the MBI scores across the subjects in all rehabilitation training tests (Figure 10). The quantified results displayed that there was no significant difference of the MBI scores between the passive and active training modes, while the sensory feedback, especially multiple-modality (visual-force) feedback, could effectively decrease the mental burdens of the subjects. It demonstrates that the subjects are more confident to perform robot-assisted training with multiple-modality feedback although maybe not the most effective training strategy. Moreover, despite all subjects agreed that the current training strategies and the training tasks were essential to upper limb functional rehabilitation, the actual rehabilitation training should patient-tailored according to different levels of upper limb dysfunction.

### 4.4 Implications, limitations and future work

The actual robot-assisted rehabilitation effects were highly related to the stroke phase of patients, dose-matched training protocols and evaluation indexes (Doumen et al., 2023). The current study quantitatively evaluated the interaction mechanism of active/passive training modes, visually-guided training tasks and multiple-modality feedback on the effects of robot-assisted rehabilitation training. The key significance lies in that it provides potential guidelines for establishing patient-specific rehabilitation protocols. From an application perspective, the visually-guided training tasks could be adopted either alone or in combination according to the individual needs of patients. BPT-visual should be applied preferably for the patients with severe upper limb motor dysfunction to obtain the maximum target muscle activation through high-intensity repetitions of passive training. UAT-visual-force is better suitable for the patients with limited motor control skills or those who have relatively higher psycho-physiological burden because of robot-assisted rehabilitation treatment. UAT-force can be used for high-intensity repetitive training on the patients with a certain level of motor dysfunction since it has a significant advantage in activating the patient’s active participation, especially for those with visual impairments. The actual performance of the multiple training strategies deserves to be comprehensively investigated by further clinical assessments.

There are still several limitations to this research. First, the narrow age range of participants (young subjects) may not fully represent the demographics typical of clinical rehabilitation populations. Second, the exclusive use of right-handed participants limits the generalizability of findings to left-handed individuals, who may exhibit distinct neurophysiological adaptations to training. Future studies should address these gaps by recruiting a broader age spectrum and including participants with diverse handedness to enhance the applicability of results to real-world clinical settings. Although the qualitative training protocols have been demonstrated on the healthy subjects, the further clinical efficacy has not been conducted on the dysfunction-matched individuals due to lack of eligible patients. In addition, it should be mentioned that we only used the myoelectric activations of the target muscles to characterize the subjects’ participation levels in training tasks. The subject’s behavioral indexes and the corresponding brain functional responses should be adopted to reflect the effects of the rehabilitation training. Effective evaluation indexes of electrophysiology such as, time-frequency and network responses of EEG, fNIRS and fMRI, should be explored in further investigations.

In the follow-up work, a batch of patients with varying degrees of upper limb dysfunction will be recruited to test the long-term rehabilitation effects of the current training protocols, and compared to the therapist’s manual scale assessment, i.e., Fugl-Meyer Assessment for Upper Extremity (FMA-UE). Quantifiable electrophysiological indexes of brain functional responses (i.e., time-frequency responses and multiple-region activation networks of EEG), which can represent the degrees of integration mechanism of visual, auditory and tactile feedback, a patient’s rehabilitation recovery, and allocation of cognitive resources, will be comprehensively investigated to objectively evaluate the merits and shortcomings of the robot-assisted rehabilitation treatment.

## 5 Conclusion

In conclusion, the current results suggested that unilateral/bilateral training with optimal virtually-guided tasks and visual/force feedback could be effectively used to robot-assisted rehabilitation training in patients with upper limb dysfunction. The commonly-used training (UPT, BPT, and UAT), typical visually-guided tasks and visual/force feedback were essential for establishing patient-tailored rehabilitation protocol. The proposed effective training strategies could be applied to the patients with different degrees of upper limb dysfunction.

## Data Availability

The datasets presented in this article are not readily available because the data are available from the corresponding author on reasonable request. Requests to access the datasets should be directed to GC, ghchai99@nimte.ac.cn.
